# Measuring the Distribution of Spitefulness

**DOI:** 10.1371/journal.pone.0041812

**Published:** 2012-08-15

**Authors:** Erik O. Kimbrough, J. Philipp Reiss

**Affiliations:** 1 Department of Economics, Simon Fraser University, Burnaby, British Columbia, Canada; 2 Department of Economics (AE1), Maastricht University, Maastricht, Limburg, The Netherlands; CSIC-Univ Miguel Hernandez, Spain

## Abstract

Spiteful, antisocial behavior may undermine the moral and institutional fabric of society, producing disorder, fear, and mistrust. Previous research demonstrates the willingness of individuals to harm others, but little is understood about how far people are willing to go in being spiteful (relative to how far they could have gone) or their consistency in spitefulness across repeated trials. Our experiment is the first to provide individuals with repeated opportunities to spitefully harm anonymous others when the decision entails zero cost to the spiter and cannot be observed as such by the object of spite. This method reveals that the majority of individuals exhibit consistent (non-)spitefulness over time and that the distribution of spitefulness is bipolar: when choosing whether to be spiteful, most individuals either avoid spite altogether or impose the maximum possible harm on their unwitting victims.

## Introduction

The Stanford Prison Experiment revealed the startling facility with which individuals lapse into antisocial and sadistic behavior when given the means and opportunity [1], [2]. Given the chance, individuals may readily abandon the peaceable character common to their daily lives and systematically and brutally mistreat others. Similar destructive tendencies are evident following breakdowns in traditional mechanisms of social control, as in the looting and indiscriminate vandalism that often follow in the aftermath of natural disasters and political demonstrations [3]. Under less extreme conditions, scattered acts of spitefulness nevertheless occur frequently, from locals pettily misleading tourists to children bullying their smaller peers.

Explanations for these (mis-)behaviors variously emphasize the importance of situational factors (e.g. dehumanization, differences in relative power, anonymity, action unobservability) [1], [2], [4]–[6] and individual characteristics such as personal history and personality 7–9. However, evidence from twin studies also suggests that anti-social behavior has a strong genetic component [10].

When we call an action “spiteful”, we mean that it directly imposes harm on another and provides no immediate benefit to the spiteful actor. Our notion of spite differs from that typically employed by evolutionary biologists in that the latter require the spiter to undertake an (expected) cost when reducing the relative fitness of the other [11], [12]. Costly spite has been observed in some non-human species, e.g. social insects [13] as well as in humans [14]–[18].

However, many empirical studies of spiteful behavior with human subjects suffer from identification problems. In the case of the classic social-psychology research [1], [2], it is unclear to what degree spiteful actions are undertaken as a result of implicit or explicit experimenter demand as opposed to individual desire to do harm. In ultimatum games, there is debate over the motivations for decisions to reject non-zero offers (see e.g. [19]). However, since the decision to reject an offer is all or none, even if rejections represent spite (as claimed in [16]), it is still impossible to measure the *extent* of spitefulness. In the public goods games reported in [17], it is unclear whether the observed behavior is spite or merely an attempt to signal current dissatisfaction with the goal of promoting future cooperation. Recent experiments on costly ‘antisocial punishment’, in which some individuals actively punish cooperative others has been observed in a broad range of cultures and environments, improve upon these studies because they directly measure the extent to which individuals are willing to endure costs to impose harm on others [15], [20], and recent models suggest that such behavior may be a result of selection [21]. However, in environments where spite is costly, individuals face an unobservable tradeoff between the costs and benefits of being spiteful. The presence of this tradeoff complicates inference about spiteful strategies because measured spite will be sensitive to the relative costs of spite to the spiter and to the target.

One advantage of studying spite in auctions, particularly second price auctions, is that spite can be measured in the intentional increase of the price that another bidder must pay. This element was recognized in previous studies of spite in auctions. For example, [22] report an experiment in which subjects in two-bidder, asymmetric second-price and ascending bid auctions with complete information on other bidders' values. They observe that lower value subjects overbid their values more frequently than higher value subjects. Similarly, in the auctions reported in [18], overbidding one's value may be explained by spite, but in both experiments, subjects submit bids without knowledge of the current high bid so that these bids still imply some risk of winning the auction. This feature inhibits measurement of spite as some overbidding may also be explained by judgment errors and/or a desire to win the item, regardless of cost. See also, [23] for an earlier paper modeling spiteful preferences in auctions which potentially explains overbidding in first-price auctions.

Evidence from mosquitos indicates that when the costs are borne only by the target, spiteful behaviors can and will persist [24]. In our experiment, spite is also costless to the spiter, so that we can directly observe the underlying willingness to do harm.

Although (non-)spitefulness is a prominent behavioral pattern, little is known about how *observed* harm compares to the maximum harm that could have been done or to what degree (non-)spitefulness is stable within individuals over repeated trials. To isolate these aspects of spite, we report an incentivized laboratory experiment, holding situational factors constant, in which we can observe spiteful behavior, and we ask: when individuals have means and opportunity to anonymously harm others at zero personal cost, what is the prevalence, extent and individual consistency of spitefulness? Is there heterogeneity of spitefulness across individuals?

The remainder of the paper is organized as follows: In the next section, we introduce the experimental design. The following sections report our results and a discussion of their implications. Then, we provide a detailed description of our experimental procedures, and Appendix S1 provides a copy of our instructions.

### Experimental Design

To generate opportunities to observe spiteful behavior and measure the extent of spitefulness, subjects participate in a sequence of 16 market periods in each of which they attempt to buy a single unit of a fictitious item. In each of the 16 periods, one unit of supply is available for purchase in a two-stage auction, and unlimited supply of an identical item is available at a fixed price after the auction ends. In the first stage of the auction, bidders are informed about their value and the fixed price at which they can buy after the auction, and they submit an initial bid for the auctioned item. In the second stage, everyone is informed about the highest initial bid, and subjects submit their final bids. The item is allocated to the highest bidder at a price equal to the *second highest* final bid, so our auctions represent stylized versions of those employed by the auction website eBay [25], [26]. Subjects must submit a final bid, and it must be at least as high as their initial bid, but they are permitted to resubmit the same bid. Those subjects who do not purchase an item at auction can then purchase it in the aftermarket at the fixed price.

We call a bidder *spiteful* if she increases the final auction price with no intention of winning the auction. By submitting a final bid higher than her own initial bid but also lower than the *highest* initial bid, a spiteful bidder can increase the price paid by the winning bidder (thereby reducing that person's earnings) at no cost to herself. Typically in auctions, spiteful bidding may be inhibited by bidders' uncertainty about the current highest bid. Without this information, making a bid with the goal of driving up the auction price entails some risk of winning the auction and paying the high price as, e.g., on eBay. We eliminate this risk of unintentionally winning the auction by announcing publicly - just before bidders submit their final bids - the highest bid submitted in the first stage of the auction. This feature of our experiment not only enables us to observe spiteful behavior *per se* but also allows us to quantify the extent of feasible spitefulness: the maximally spiteful bid is the largest bid that avoids winning the auction. This crucial element distinguishes our setup from [22], since bidders in their experiments know others' values but remain uncertain about their *bids* until the auction ends. Thus, a spiteful bid in their environment still entails some risk and does not permit measurement of relative spitefulness.

We informed subjects only about the highest initial bid and *not* the second-highest initial bid (which would determine the price if the auction ended then), so they could not differentiate between auction prices that were set by competitive bidding in the initial stage or by spiteful bidding in the final stage. This prevented subjects from conditioning their behavior on having been harmed because they could not even know if they were the object of spite. This design element rules out, e.g., positive and negative reciprocity, which were argued to drive behavior in [22].

After reviewing the highest initial bid, a subject who is not the high bidder in the first stage can choose whether to engage in spite (by driving up the price) or not (by keeping his bid constant), but he may also choose to increase his bid above the highest initial bid in an attempt to win the auction. To measure the extent of spitefulness, it is essential that subjects frequently encounter a decision where the latter action is undesirable, or else we would rarely observe a choice between spiteful and non-spiteful bidding. To increase the likelihood that a subject may decide whether to be spiteful, we provide each bidder not winning the auction with the opportunity to buy an item - identical to the auctioned item - at a fixed price after the auction. Thus if the highest initial bid meets or exceeds the (expected) fixed price, a bidder should never choose to buy at auction and will instead wait to buy the identical item later on, but nevertheless, each bidder has to submit a final bid.

We collected all initial bids before providing subjects with any feedback and before beginning the second stage of any auction. This removes the possibility that spitefulness can be justified as a way to teach other subjects that submitting unreasonably high initial bids can be a costly mistake. We provided feedback on the auction outcome after all bidders submitted final bids. This provided subjects with many opportunities to observe how spiteful bids affected the winning bidders' earnings so that repeated submission of spiteful bids by the same subject cannot be dismissed by inadvertency.

We designed this experiment as part of a research program on price formation in auctions, unrelated to spiteful behavior. For this reason, our design incorporates an individual choice “real effort” task between the auction stages and an extra decision, prior to submitting the final bid, in which subjects may choose whether to recall the fixed price at which they may purchase in the aftermarket or to earn money in the “real effort” task.

Each experimental session consists of three stages: an “Opening” stage in which they submit initial bids in the auction; an “Effort” stage in which they earn money by completing a real effort task; and a “Closing” stage in which subjects submit a final bid in the auction and then subjects who are unable to purchase an item at auction.

In the “*Opening*” stage, each subject submits a sequence of 16 initial bids, one for each auction. For each auction, subjects observe their induced value for the item and the fixed price at which they will later be able to purchase. After receiving this information for the first auction, they submit their first bid. Then the process repeats until they have submitted initial bids for all 16 auctions.

Following the initial bids, subjects enter the “*Effort*” stage. Here each subject participates in 3, two-minute periods of a real-effort ‘slider task’ [27] with a break of one minute in between. Subjects observe a screen with 48 sliders, each representing a scale from 0 to 100. The sliders are initially set to “0”, and subjects receive a payment 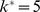 for each slider that they set to “50” by the end of the two minutes. (We expect subjects being capable of correctly placing 15–20 sliders in a two-minute period leading payments of 1.50–3.00 EUR over the three tasks.)

At the end of the Effort stage, subjects enter the third and final stage of the session in which each auction ends in the same sequence in which the subject submitted bids in the *Opening* stage. The “*Closing*” stage is divided into two sub-stages *for each auction*, which we call the “*Market*” and the “*Aftermarket*”. In the *Market* sub-stage, subjects observe their bid and whether they are currently the highest bidder. Then, they may submit a new bid if they desire. However, if they want to recall the fixed price before making their bid, they must agree to forgo the opportunity to participate in another minute of the slider task. Hence, by varying the value of *k* in the slider task, we vary the opportunity cost of recalling the fixed price, and we can observe the effect of opportunity cost on the probability of overbidding in the auction. We do not discuss the findings related to this hypothesis here, but we are currently writing a companion paper that addresses these issues.

After each subject submits a new bid (or resubmits the original bid), those subjects who did not choose to observe the fixed price participate in one minute of the slider task in which each correctly placed slider yields a return of 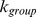. Those who choose to forgo the slider task must wait quietly. At the end of the minute, the auction immediately concludes with the highest bidder paying the second highest bid. Each subject learns whether they were able to purchase the good at auction, and those subjects who were unsuccessful are then given the opportunity to purchase in the *Aftermarket* sub-stage. In the *Aftermarket*, the fixed price is revealed to all the remaining buyers at no cost, and subjects simply choose whether or not to buy at the revealed price. At the end of the first aftermarket, the second auction enters the *Closing* stage, and so on until all the auctions have closed.

Each auction consists of 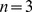 bidders chosen from a matching group of size 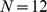. For each auction, each subject receives a randomly drawn integer value, 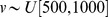, and we draw the fixed price, 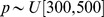, so that buyers will always buy in the aftermarket even if they are unable to buy at auction. Thus, we can observe spite in the absence of concerns about being unable to purchase an item. Over the sequence of 16 auctions, all four groups face the same values and fixed price in each auction. Our design also varies the value of *k* in the slider task (though this variation does not matter for the results we report here). Specifically, in each auction, one group of 3 bidders faces each of 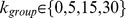. We rematch groups to ensure that each bidder faces each value of 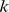 four times.

Subjects are rematched across auctions and receive no additional information about the other bidders in their auction. The bidder's role is framed as that of a seller facing the opportunity of buying several commodities in the auctions for resale to the experimenters, one commodity per auction. One advantage of this framing is that it better motivates participation in multiple auctions. In the instructions, we inform subjects that they will learn the fixed price in the *Opening* stage and that they “will have the option to review the fixed price before submitting a new bid in the *Closing* stage.” This language avoids the implication that the opportunity is *freely* available without directly revealing our treatment variation. We also inform subjects that “if [they] are unable to buy the item in the auction, [they] will be able to purchase an identical item during the *Aftermarket*.” See Appendix S1 for a complete set of instructions.

## Results

**Figure 1:**
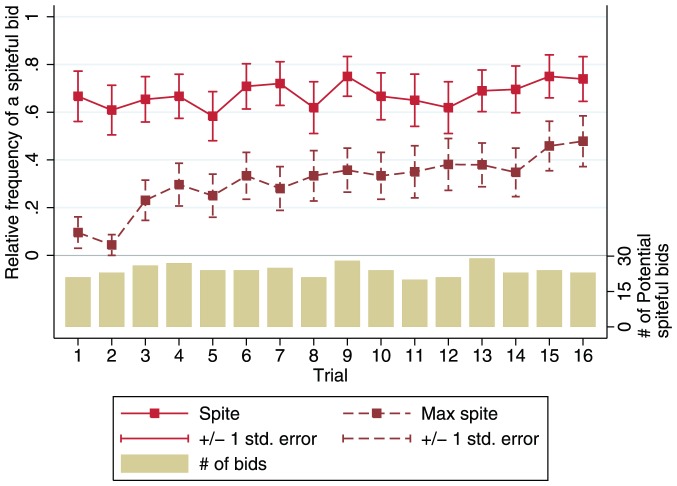
Time series of the relative frequencies of spiteful and maximally spiteful bids, conditional on bids being potentially spiteful. The solid line indicates the observed relative frequency of subjects making a spiteful bid in each trial, and the dashed line indicates the observed relative frequency of a maximally spiteful bid. Capped spikes display the standard errors of the mean in each trial. The subgraph displays the number of opportunities to observe a spiteful bid in each trial, and suggests that the relative frequency of observing spite is not related to the number of opportunities to be spiteful.

We define *potentially spiteful* bids as all final bids that were not directed at winning the auction (i.e. were lower than the highest initial bid). Subjects submitted potentially spiteful bids in 383 out of 768 instances, so we have ample opportunity to identify spite. We call a final bid *spiteful* if it is *both* greater than the bidder's own initial bid and less than the highest initial bid so that it necessarily increases the price paid by the auction winner. We call a final bid *maximally spiteful* if it is exactly one bidding increment less than the highest initial bid (e.g. if the highest initial bid was 100, a maximally spiteful bid would be 99). Such a bid maximizes the loss a spiteful bidder can impose on the auction winner without risk of winning the auction. We observe abundant spite as 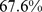 of all potentially spiteful final bids are actually *spiteful*. Furthermore, 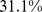 of potentially spiteful bids are maximally spiteful. The frequency of observing a spiteful bid is roughly constant over trials, but the frequency of maximally spiteful bids is increasing (Figure 1).

**Figure 2:**
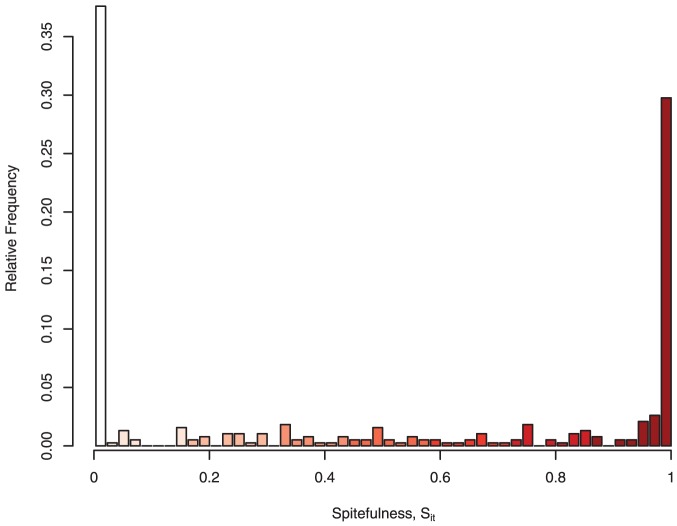
Histogram of measured spitefulness (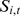), pooled over all subjects and trials. The height of each bar represents the relative frequency of observing spitefulness in each of 50 intervals of length 0.02. Nearly 70% of the weight of the distribution is in the extreme values zero and one, indicating that, given the opportunity, subjects are either maximally spiteful or not spiteful at all.

To facilitate inter- and intra-subject comparisons of the *extent* of spitefulness, we compute the share of observed harm imposed (price increase) by subject 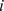 in trial 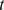 out of the maximum possible harm in that trial. Denote the initial bid and final bid by 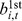 and 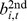 and the highest initial bid by 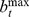. Spitefulness is defined as the actual bid increase divided by the maximum possible bid increase that implies no risk of winning the auction,
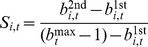
conditional on a subject not attempting to win the auction, so that 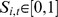. The distribution of spitefulness indicates that we not only observe spiteful bids with striking frequency, the distribution of spitefulness is also highly skewed to both tails, indicating that most bids are either not spiteful at all or maximally spiteful (Figure 2). Note that for a bidder who has decided *not* to try to win the auction, any bid less than the first stage high bid is, strictly speaking, a weak best response. However, if subjects were adopting such a strategy, we would expect them to choose each value between their current bid and the current high bid with equal probability. Clearly from the u-shaped distribution of spitefulness in Figure 2, this is not the case.

**Figure 3:**
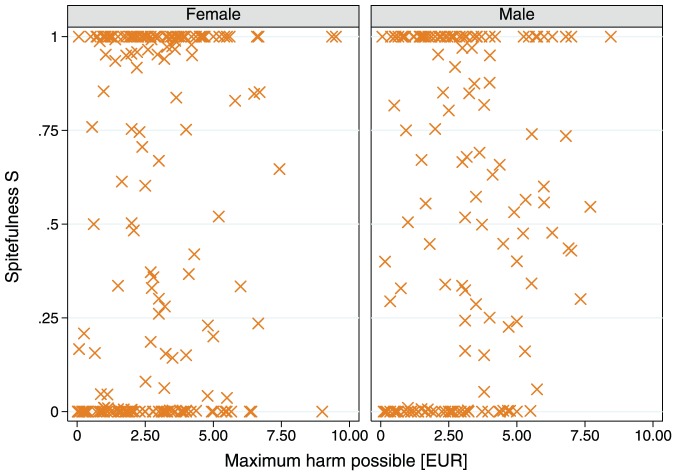
The effects of the magnitude of maximum harm possible and gender on spitefulness. The panels present the data separately for males and females. Each data point represents one observation of spitefulness relative to the magnitude of maximum harm possible.

Our measure of spitefulness 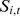 does not distinguish between a maximally spiteful bid that raised the final price by 2 and another that raised the final price by 200, although the extent of spitefulness may depend on the maximal harm that can be imposed. However, the data suggest that the decision to be maximally spiteful, as measured by 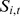, is independent of the potential harm done (Figure 3). We present the data separately for men and women to control for a potential gender effect as there is mixed evidence on gender differences in antisocial behavior [10], [28]. Our data in both panels suggest the absence of a gender effect.

Panel regression analysis (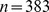) confirms the absence of both magnitude and gender effects. We regress spitefulness on the magnitude of potential harm, a gender dummy, an interaction between female and magnitude of potential harm, the aftermarket price, and a time (trial) trend along with a constant. The estimates show that spitefulness is affected by neither the level of possible harm (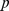-value 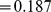) nor by gender or the interaction term (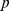-values 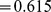 and 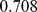). We include the aftermarket price because it may potentially affect spitefulness. For example, when the aftermarket price is high, those bidders who do not buy in the auction may want to raise the price paid by auction winners so that their earnings are not substantially different. Thus, [29] observe that in a modified dictator game, when individuals receive low offers, some subjects are willing to incur a small cost to substantially reduce the payoff of the other. However, we find that spitefulness is unaffected by the aftermarket price (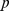-value = 0.357); full regression output is available in Table 1. Consistent with the increase in spitefulness over time (Figure 1), the estimated time trend is positive and highly significant (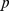-value 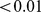).

**Table 1:** Random effects estimation of relative spitefulness 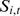.

Independent Variable	Coefficient	Rob. Std. Err.	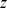	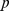 -value	95% conf. interval
Maximum Possible Harm	0.00015	0.00011	1.32	0.187	[−0.00007, 0.00036]
Aftermarket Price	−0.00020	0.00022	0.92	0.357	[−0.00063, 0.00023]
Female	−0.05767	0.11480	−0.50	0.615	[−0.28267, 0.16733]
Female (Max. Harm.)	−0.00005	0.00014	−0.37	0.708	[−0.00033, 0.00022]
Trial	0.02105***	0.00652	3.23	0.001	[0.00827, 0.03383]
Constant	0.38353***	0.11886	3.23	0.001	[0.15058, 0.61649]

Significance levels are denoted by: 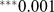, 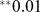, 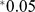. The random effects error structure is included for individual subjects to control for repeated measurement. A positive and significant coefficient of Trial (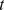) indicates increasing spitefulness over time. Note also the insignificant coefficients on Maximum Harm, Aftermarket Price, Female and their interaction.

Finally, we examine the heterogeneity of spitefulness between and within individuals. While 5 subjects are maximally spiteful at every opportunity (i.e. 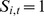 whenever spite was possible), 6 of them are never spiteful (i.e. 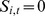 whenever spite was possible). Similarly, we find that 13/45 subjects are maximally spiteful at least 50% of the time, and 14/45 subjects are not at all spiteful at least 50% of the time. This is markedly similar to the observed distribution of spitefulness in Figure 2.

**Figure 4:**
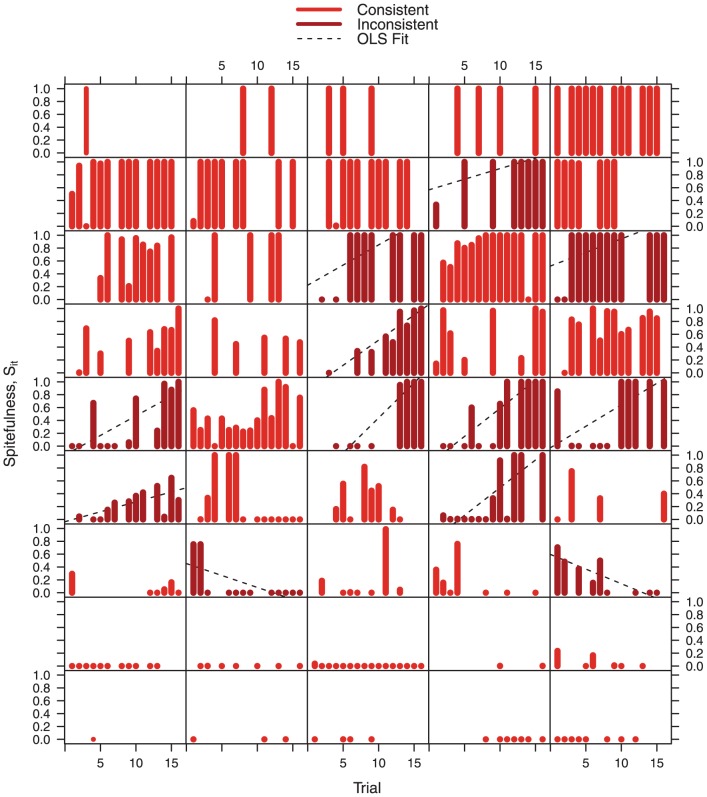
Barplot time series of individual spitefulness ( 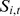
 **).** Each panel displays the spite data for a single subject. Panels are sorted from top to bottom by average spitefulness. Each bar represents the measured spitefulness of a subject in the indicated trial; if the bar has the length of a dot, the subject had the opportunity to act spitefully but chose to be non-spiteful. Each dashed line shows a linear fit to the data for subjects classified as behaviorally inconsistent, i.e. if the estimated coefficient of trial (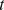) is insignificant at a level of 5%. Out of 48 subjects, 45 had the opportunity to submit spiteful bids. The data reveal both considerable heterogeneity in spitefulness across individuals and consistency within individuals.

More generally, many subjects display striking consistency in their level of (non-)spitefulness (Figure 4). To further evaluate individual consistency, we estimate a simple linear regression, separately for each subject, where the dependent variable is spitefulness 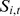 and the independent variable is trial 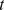. We classify a subject as behaviorally inconsistent if the regression yields a significant estimate of the trial coefficient. By this criterion 73.3% of our subjects display consistent levels of (non-)spitefulness. Note that the individual stability of spitefulness appears to be inconsistent with the significant aggregate time trend noted in Figure 1 and Table 1; the reason we observe increasing average spitefulness over time is that, among the individuals whose level of spitefulness is *not* consistent over time, 10 out of 12 show an increasing trend. This creates an increase in the aggregate level of spitefulness, despite the stability of most individuals.

## Discussion

**Figure 5:**
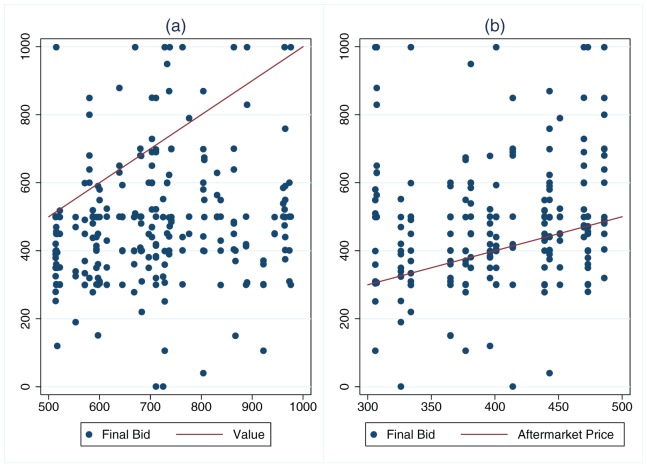
Scatterplots of final bids submitted by potentially spiteful bidders. Panel (a) shows the relationship between potentially spiteful final bids and values, and panel (b) shows the relationship between such bids and the aftermarket price. Each point represents a single bid, and the lines provide a reference showing where the bid equals each value or aftermarket price.

One potential concern with our method of measuring spite is that some bids which we label spiteful may result from alternative bidding strategies. For example, bidders who discover that the initial high bid is greater than their value have a weak best response to bid their value, and if their initial bid is less than their value, this strategy will produce a positive measure of spitefulness. Similarly, bidders who know the posted aftermarket price have a dominant strategy to bid the minimum of their value and the posted price; here too, for a sufficiently low initial bid, a final bid that follows this strategy will be measured as spiteful. Figure 5a provides a scatter plot of potentially spiteful bidders' final bids against their values, and Figure 5b plots potentially spiteful bidders' final bids against the aftermarket price. Neither values nor aftermarket prices appear to account for observed bids.

Our experiment places subjects in repeated situations in which they choose whether to spitefully harm an anonymous other at zero cost and in which spiteful acts are not revealed as such during or after the interaction. Because we have access to information not only about how much harm was imposed, but also how much harm *could have been* imposed, we are able to directly measure each individual's level of spitefulness. In this setup we find that spitefulness widely prevails, but its distribution is bipolar; typically we observe either zero or maximum spitefulness. The shape of the distribution is neither accidental nor generated by arbitrary behavior on the part of subjects. Instead the large majority of subjects exhibit consistent spitefulness across trials suggesting the existence of a stable individual characteristic of (non-)spitefulness, conditional on circumstances.

## Methods

### Ethics Statement

All experiments were conducted with the informed consent of 48 healthy adult subjects who were free to withdraw from participation at any time. Only individuals who voluntarily entered the experiment recruiting database were invited, and informed consent was indicated by electronic acceptance of an invitation to attend an experimental session. The experiments were conducted following the peer-approved procedures established by Maastricht University's Behavioral and Experimental Economics Laboratory (BEElab). Our study was approved by the BEElab at a public ethics review and project proposal meeting that is mandatory for all scholars wishing to use the BEElab facilities.

### Experimental Procedures

The experiments were conducted at the BEElab of Maastricht University with 48 students. Subjects' decisions were fully incentivized using Experimental Currency Units (ECUs). Their ECU-profits were converted to EUR at a rate of 100 ECU = 1 EUR and these from two randomly selected auctions were paid to them in cash at the end of the experiment. In order to avoid the influence of wealth effects, at the end of the experiment, we randomly select two auctions for each subject for payment. Then subjects receive private cash payments including a 4 EUR payment for arriving to the experiment on time, their earnings from the Effort stage, and their earnings from the two randomly selected auctions (including what they earned in the slider task, if they participated).

In total we ran 4 sessions of 12 subjects drawn from the undergraduate population of Maastricht University (Average age = 22.6, 46% Female). On average, subjects earned 15.32 EUR for a 60-minute session ranging from a low of 6.93 EUR to 26.65 EUR, including show-up payment. All sessions were conducted in May 2011.

Our experiment was programmed using the z-Tree software package [30]. Some graphics and data analysis were performed using 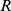, an open-source statistical software [31].

## Supporting Information

Appendix S1 Instructions that were provided to experiment subjects.
(PDF)
