# Supplementary material for: Measuring the Distribution of Spitefulness
Source: PLoS One. 2012 Aug 15;7(8):e41812. doi: 10.1371/journal.pone.0041812 (PMC3419693; doi:10.1371/journal.pone.0041812)
Supplement: Appendix S1 — Instructions that were provided to experiment subjects. (PDF) [file pone.0041812.s001.pdf]

Appendix S1: Experiment Instructions

General information

You are now participating in a decision making experiment. If you follow the instructions carefully, you can earn a considerable amount of money depending on your decisions and the decisions of the other participants.

This set of instructions is for your private use only. During the experiment you are not allowed to communicate with anybody. In case of questions, please raise your hand. Then we will come to your seat and answer your questions. Any violation of this rule excludes you immediately from the experiment and all payments. The research organisation METEOR has provided funds for conducting this experiment.

During the experiment the outcome of your decisions will be measured in ECU (Experimental Currency Units) instead of Euro. We will convert your total earnings into Euro at a rate of 100 ECU = 1 Euro at the end of the experiment and pay you in cash privately.

Outline of the Experiment

This experiment consists of 16 auctions of fictitious items, each of which will have some value to you; you can think of your value as the amount of money (in ECU) that the experimenter will pay you for the item if you buy it. So, if you have a value of 800 ECU for the item, and you buy it in the auction, you will earn 800 minus the price (800 - P) ECU.

In each auction, your value will be a random number between 500 and 1000 ECU. Each value is equally likely. You will have a new random value in each auction.

There are three (3) bidders in each auction, and the two (2) other people in each auction will change randomly. Other people also receive random values between 500 and 1000 ECU for each item, and all values are determined independently of one another. You will only know your own value, and the other people will only know their own values.

At the end of each auction, the person who submitted the highest bid receives the item and pays for it a price equal to the second highest bid. If there is a tie for the high bid, the item is assigned to one of the bidders by chance, and the price paid is equal to the bid.

If you do not submit the highest bid, so that you do not receive the item in the auction, you can buy an identical item after the auction at a fixed price between 300 and 500 ECU, called the aftermarket price.

Anyone who did not buy the item in the auction can buy a unit of the item at the aftermarket price. This item is identical to the auctioned item so that you have the same value for it as for the auctioned item.

So, if the aftermarket price is 400 ECU and you choose to buy the item after the auction, you would receive 800 - 400 = 400 ECU.

Each of the 16 auctions is divided into 3 stages:

(1) *Opening Stage* each person submits a first bid for the item.

(2) *Task Stage* each person participates in a task in which they can earn money by manipulating objects on the computer screen.

(3) *Closing Stage* each person submits a second and final bid in the auction, and this bid cannot be smaller than their first bid. Everyone will learn whether they bought item at auction, and the people who did not buy at auction will have the opportunity to buy an identical item at the aftermarket price.

The instructions that follow will give you detailed information about stage 1. After completing stage 1, you will receive detailed instructions for stage 2 and stage 3.

At the end of the experiment, we will pay you the total of your earnings from the task in stage 2 plus your earnings from 2 randomly chosen auctions. In addition, you begin the experiment with an initial endowment of 400 ECU for arriving on time.

Stage 1: The Opening Stage

To begin the experiment, you will participate in the Opening stage for all 16 auctions.

In the Opening stage of each auction, you will see your value for the item and the aftermarket price at which, after the auction, you will be able to buy an identical item if you are not the high bidder in the auction.

After reviewing this information, you submit a first bid for the item. Your bid can be thought of as being the price that you want to pay for the item in the auction.

In the center of the screen, you will see a box in which you can type your first bid. Once you type in a first bid that you are happy with, you will click the button labelled "Submit First Bid".

At the same time, the other people in your auction will submit their own first bids. Once all bids are submitted for the first auction, the Opening stage will begin for the second auction.

In the second auction, you will again see your value and the aftermarket price for a new item, and as before you will submit a first bid.

This process will repeat until you have submitted a first bid for all 16 auctions.

Later, in the 3rd stage of the experiment, you will see the highest first bid in each of the 16 auctions, and you will have the opportunity to submit a second and final bid in each auction. Note, your final bid cannot be smaller than your first bid, but you can submit the same bid again if you wish.

Stage 2: The Slider Task Stage

The Slider Task stage allows you to earn money from a computerised task.

You will now participate in 3 rounds of an identical task lasting 120 seconds per round with a break of 60 seconds between each round. Rounds 1 and 2 allow you to practice the task and will not affect your earnings. The score that you will have obtained in round 3 will be used for determining your total earnings from the Slider Task Stage.

The task will consist of a screen with 48 sliders. Each slider is initially positioned at 0 and can be moved as far as 100. Each slider has a number to its right showing its current position.

You can use the mouse or the keyboard's arrow keys in any way you like to move each slider. To use the arrow keys you have to select the slider that you want to adjust first by clicking it with the mouse. You can readjust the position of each slider as many times as you wish. You adjust the sliders to earn points. Specifically, your score in the task will be the number of sliders positioned at exactly 50 at the end of the 120 seconds.

Your score in round 3 will be converted to ECU at a rate of 1 point = 5 ECU. For example, if after 120 seconds you have correctly placed 6 sliders at 50, then you have earned 6 points, which is converted to 30 ECU and will be paid to you at the end of the experiment.

Thus your Slider Earnings are equal to (Your Score in round 3 x 5 ECU)

Are there any questions?

Stage 3: The Closing Stage

The Closing stage of the 16 auctions consists of two parts, the Market and the Aftermarket. In the Market part, each person submits a second and final bid for each of the 16 auctions. Before submitting your final bid, you will be informed of your value, your first bid, and the highest first bid. After reviewing this information, you will have the option to either:

1) Review the aftermarket price before submitting your bid

OR

2) Participate in another 30-second round of the Slider Task to earn ECUs.

If you choose to review the aftermarket price, you will not be able to participate in the Slider Task and you will have to wait quietly for 30 seconds. During the 30 seconds you will be able to see the aftermarket price at which the item will be available in the Aftermarket part.

On the other hand, if you choose to participate in the Slider Task, you will earn additional ECU based on your score from correctly adjusting sliders to 50, but you will not be able to review the aftermarket price before submitting your final bid. The amount of ECU that you receive per correctly adjusted slider will change over the course of the 16 auctions and you will be informed about it before you choose to participate, or not to participate - in the task.

At the end of 30 seconds, everyone will submit a final bid. On the bid submission screen, everyone will see their value, their first bid, and the highest first bid. After you review the available information, type in your final bid and click the button labelled "Submit Final Bid".

After everyone in your auction submits a final bid, you will be informed of the outcome of the auction. Remember, the person who submits the highest final bid receives the auctioned item and pays a price equal to the second highest final bid. Therefore, your Market Earnings for each auction are computed as follows:

- if you are the high bidder:
  – Your Market Earnings = (Your value - 2nd highest final bid) ECU
- if you are not the high bidder:
  – Your Market Earnings = 0 ECU

If you are the high bidder so that you receive the auctioned item, then your income in this round is equal to your Market Earnings and you do not participate in the Aftermarket. However, if you are not the high bidder, then you will have the opportunity to buy an identical fictitious item in the Aftermarket as described in the following.

In the Aftermarket, you will be reminded of your value for the item (which is exactly equal to your value for the auctioned item) and the aftermarket price. If you want to buy the item at the aftermarket price, then you click the button labelled "Buy".

Your Aftermarket Earnings are determined as follows:

- if you choose to buy the item in the aftermarket:
  – Your Aftermarket Earnings = (your value - the aftermarket price) ECU
- if you choose not to buy the item in the aftermarket:
  – Your Aftermarket Earnings = 0 ECU

At the end of the Aftermarket, the Closing stage is over and the auction is complete. Then we repeat the Closing stage until all 16 auctions are completed.

Your Earnings

Any auction can end in exactly six ways, and your Auction Earnings for an individual auction for each of these cases are as follows:

1. You win the item in the auction, and you participate in the Slider Task
   Auction Earnings = (your value - 2nd highest final bid + Slider Task Earnings) ECU

2. You win the item in the auction, and you do not participate in the Slider Task
   Auction Earnings = (your value - 2nd highest final bid) ECU

3. You do not win the item at auction, you participate in the Slider Task, and you choose to buy an item in the Aftermarket.
   Auction Earnings = (your value - aftermarket price + Slider Task Earnings) ECU

4. You do not win the item at auction, you do not participate in the Slider Task, and you choose to buy an item in the Aftermarket.
   Auction Earnings = (your value - aftermarket price) ECU

5. You do not win the item at auction, you participate in the Slider Task, and you choose not to buy an item in the Aftermarket.
   Auction Earnings = Slider Task Earnings ECU

6. You do not win the item at auction, you do not participate in the Slider Task, and you choose not to buy an item in the Aftermarket.
   Auction Earnings = 0 ECU

Total Earnings

At the end of the experiment, we will randomly draw 2 out of the 16 auctions for payment and sum your earnings from the 2 auctions. If your auction earnings are positive, then your ECU balance increases by this amount. If your auction earnings are negative, you made a loss and your ECU balance decreases by the amount of the loss. If your auction earnings are 0 ECU, then your ECU balance does not change.

Finally, we will add your earnings from the Slider Task stage to your ECU balance and we will convert it to Euro at a rate of 100 ECU = 1 Euro. When the experiment is over, please wait quietly until we call you for payment.
